# Incidence and Predictive Factors of Acute Kidney Injury After Major Hepatectomy: Implications for Patient Management in Era of Enhanced Recovery After Surgery (ERAS) Protocols

**DOI:** 10.3390/jcm14155452

**Published:** 2025-08-02

**Authors:** Henri Mingaud, Jean Manuel de Guibert, Jonathan Garnier, Laurent Chow-Chine, Frederic Gonzalez, Magali Bisbal, Jurgita Alisauskaite, Antoine Sannini, Marc Léone, Marie Tezier, Maxime Tourret, Sylvie Cambon, Jacques Ewald, Camille Pouliquen, Lam Nguyen Duong, Florence Ettori, Olivier Turrini, Marion Faucher, Djamel Mokart

**Affiliations:** 1 Department of Anesthesiology and Critical Care, Paoli-Calmettes Institute, 232 Bd Sainte Marguerite, 13009 Marseille, France; henri.mingaud@ap-hm.fr (H.M.); deguibertj@ipc.unicancer.fr (J.M.d.G.); chowchinel@ipc.unicancer.fr (L.C.-C.); gonzalezf@ipc.unicancer.fr (F.G.); bisbalm@ipc.unicancer.fr (M.B.); alisauskaitej@ipc.unicancer.fr (J.A.); sanninia@ipc.unicancer.fr (A.S.); tezierm@ipc.unicancer.fr (M.T.); tourretm@ipc.unicancer.fr (M.T.); cambons@ipc.unicancer.fr (S.C.); pouliquenc@ipc.unicancer.fr (C.P.); ettorif@ipc.unicancer.fr (F.E.); faucherm@ipc.unicancer.fr (M.F.); 2 Department of Surgery, Paoli-Calmettes Institute, 13273 Marseille, France; garnierj@ipc.unicancer.fr (J.G.); ewaldj@ipc.unicancer.fr (J.E.); nguyenduongl@ipc.unicancer.fr (L.N.D.); turrinio@ipc.unicancer.fr (O.T.); 3 Department of Anesthesiology and Critical Care, Hôpital Nord, 13015 Marseille, France; marc.leone@ap-hm.fr

**Keywords:** acute kidney injury, acute kidney failure, major liver resection, hepatectomy, ERAS, AKIMEBO score

## Abstract

**Background**: Acute kidney injury (AKI) frequently occurs following major liver resection, adversely affecting both short- and long-term outcomes. This study aimed to determine the incidence of AKI post-hepatectomy and identify relevant pre- and intraoperative risk factors. Our secondary objectives were to develop a predictive score for postoperative AKI and assess the associations between AKI, chronic kidney disease (CKD), and 1-year mortality. **Methods**: This was a retrospective study in a cancer referral center in Marseille, France, from 2018 to 2022. **Results**: Among 169 patients, 55 (32.5%) experienced AKI. Multivariate analysis revealed several independent risk factors for postoperative AKI, including age, body mass index, the use of angiotensin-converting enzyme inhibitors/angiotensin receptor blockers, time to liver resection, intraoperative shock, and bile duct reconstruction. Neoadjuvant chemotherapy was protective. The AKIMEBO score was developed, with a threshold of ≥15.6, demonstrating a sensitivity of 89.5%, specificity of 76.4%, positive predictive value of 61.8%, and negative predictive value of 94.4%. AKI was associated with increased postoperative morbidity and one-year mortality following major hepatectomy. **Conclusion**: AKI is a common complication post-hepatectomy. Factors such as time to liver resection and intraoperative shock management present potential clinical intervention points. The AKIMEBO score can provide a valuable tool for postoperative risk stratification.

## 1. Introduction

Major hepatectomies carry a high risk of mortality and postoperative complications, including acute kidney injury (AKI) [1]. AKI after abdominal surgery has received growing attention thanks to its strong correlation with early postoperative complications such as respiratory events, sepsis, prolonged hospital stays, and short-term mortality [2]. In addition, AKI also affects long-term outcomes, including chronic kidney disease (CKD) and long-term mortality [3]. The incidence of AKI following hepatectomy varies widely in the literature, ranging from 3 to 22% [4]. This variability can be attributed to the usage of non-standardized definitions of AKI and the heterogeneity of studies involving different surgical procedures [4,5]. The pathophysiological mechanisms underlying AKI after abdominal surgery appear to be related to fluid balance or hypovolemia, hepatic failure, and inflammatory responses triggered by cytokine release [6]. Numerous preoperative risk factors have been identified in previous studies and meta-analyses [7,8]. However, intraoperative risk factors have not been adequately addressed. Most studies primarily focus on crucial surgical factors [8,9], neglecting the importance of hemodynamic considerations, fluid loading, and perioperative drug management. This point is particularly critical given the growing implementation of ERAS (Enhanced Recovery After Surgery) protocols in liver resection, which entail strict fluid restriction requirements. Our study aimed to assess the incidence of postoperative AKI after major hepatectomy and identify both pre- and intraoperative risk factors. Additionally, we aimed to develop a predictive risk model for postoperative AKI and investigate the associations between postoperative AKI and 1-year mortality, as well as AKI and CKD.

## 2. Material and Methods

### 2.1. Study Design

This retrospective observational study was conducted in a cancer referral center in France (Paoli-Calmettes Institute, Marseilles, France). The study was approved by the Institutional Review Board (IRB) (AKIMEBO-IPC 2022-013) on 18 April 2022 and conducted in accordance with the principles of the Declaration of Helsinki. The IRB waived the requirement for written informed consent due to the retrospective nature of the study, in accordance with French legislation. All patients were informed about the use of their data, and none expressed opposition to participation. Data were accessed for research purposes on 25 October 2022. The dataset used for analysis was fully anonymized before access by the investigators. No information allowing for the identification of individual participants was accessible to the authors during or after data collection. All adult patients who underwent major hepatic resection, defined as a surgical resection of three or more Couinaud segments, for either oncological or non-oncological reasons, were included in the study between January 2018 and March 2022. All patients were staged using CT and MRI at least one month before surgery. If the future liver remnant volume was deemed insufficient, portal vein embolization was performed four weeks before surgery. All patients were managed according to Enhanced Recovery After Surgery (ERAS) guidelines [10]. As part of this approach, a structured prehabilitation program was implemented, including the systematic assessment and optimization of nutritional and functional status prior to surgery. In our institution, patients scheduled for major hepatectomy are routinely admitted to the intermediate care or intensive care unit (IMC/ICU) for monitoring and standardized postoperative care for at least 2 days before moving to the surgical ward in the absence of complication.

### 2.2. Definitions

AKI was defined according to the KDIGO criteria, as recommended by the last consensus statement [11], corresponding, for stage 1, to the occurrence of 1 of the following 2 items: (1) increase in serum creatinine by ≥26.5 μmol/L within 48 h; (2) increase in serum creatinine to ≥1.5 times baseline, which is known or presumed to have occurred within the prior seven days. In stage 2, patients had creatinine levels between 2 and 2.9 times baseline. In stage 3, patients had creatinine levels above 353.6 μmol/L, 3 times baseline, or required extra renal replacement therapy. We focused on using the creatinine criteria to compare with previous studies based on the findings of Joliat et al. [12], which showed that oliguria was not associated with higher postoperative complications, unlike patients with an increase in creatinine. Acute kidney disease (AKD) was defined when AKI remained >7 days [11] after an AKI event. AKD persisting > 90 days corresponded to chronic kidney disease (CKD). CKD was assessed according to KDIGO 2012 staging [13]. Glomerular filtration rate was estimated using the CKD-EPI equation based on the creatinine level. One-year mortality was defined as death from any cause one year after the day of surgery. Overall postoperative complications were assessed according to the Clavien–Dindo classification [14]. Time to liver resection was defined as the time elapsed between the onset of anesthesia and the end of the liver resection time during the surgical procedure. Postoperative acute respiratory failure (ARF) was defined clinically as tachypnea, the recruitment of accessory respiratory muscles or respiratory muscle exhaustion, arterial oxygen saturation lower than 90% in room air, pulmonary infiltrates, and the need for high-concentration oxygen or for either invasive mechanical ventilation (IMV) or non-invasive MV (NIV). According to the Sepsis 3 criteria, sepsis was defined by the presence of suspected infection (hyperleukocytosis or leukopenia, fever, positive bacteriological samples) and a SOFA score of 2 or more. Septic shock was defined as sepsis associated with persistent hypotension, requiring the use of vasopressors to maintain a mean arterial pressure (MAP) of 65 mm Hg or more, and a serum lactate level greater than 2 mmol/L despite adequate volume resuscitation [15]. Malnutrition was defined according to the criteria of GLIM, with weight loss of ≥5% in 1 month, ≥10% in 6 months, or ≥10% from usual weight, a body mass index (BMI) < 22, or sarcopenia before surgery [16]. Post-hepatectomy liver failure (PHLF) was recorded according to the Balzan criteria [17], defined as serum bilirubin > 50 μL/L and a prothrombin time < 50% of normal on postoperative day 5, with day 0 corresponding to the day of surgery. Other postoperative organ failures were assessed using the SOFA score on day 1 and day 3.

### 2.3. Intraoperative Care

A perioperative protocol based on ERAS 2016 recommendations for liver surgery was used for all our patients [10]. General anesthesia was standardized by using a controlled infusion of either remifentanil or sufentanil and propofol for each patient. Maintenance of anesthesia was achieved by administering either desflurane or sevoflurane in a mixture of air and oxygen. Orotracheal intubation was performed to manage the patient’s airway after administering neuromuscular blockade with cisatracurium. Intraoperative analgesia combined spinal anesthesia with morphine (300 μg) and the following intravenous drugs: ketamine at 0.5 mg/kg at induction and then 0.15 mg/kg/hour, a target-controlled infusion of remifentanil, and a continuous infusion of lidocaine at a rate of 1.5 mg/kg/h, preceded by a 1 mg/kg bolus at induction according to ideal weight. Dexamethasone was systematically used for preventing nausea and vomiting. Multimodal analgesia was administered at the end of the operation and included paracetamol, nefopam, and a non-steroidal anti-inflammatory drug if there were no contra-indications. Antibiotic prophylaxis was administered according to the recommendations of the French Society of Anesthesia and Intensive Care [18]. Intraoperative fluids were limited to 1 mL/kg/h during the time elapsed between the onset of anesthesia and the end of the liver resection (time to liver resection), in the absence of shock or obvious preload dependency-related hemodynamic instability. Changes in stroke volume were estimated using transesophageal Doppler whenever possible, or by measuring changes in pulse pressure. Central venous pressure was not monitored. Blood volume was then optimized from the end of transection to the end of the postoperative period by performing vascular filling tests of 250 mL of crystalloid using a 50 mL syringe on the filling line. The main strategy was to stop vascular filling as soon as stroke volume variations of <10% were obtained, using esophageal Doppler intraoperatively or echocardiography in the intensive care unit [19]. As a routine vasopressor support, we used norepinephrine via an electric syringe pump with a target mean arterial pressure above 65 mmHg, first micro-diluted (0.010 mg/mL), then with a high concentration (0.5 mg/mL) if necessary. Major hepatectomy was performed either via laparotomy through a Makuuchi J-shaped incision or by a laparoscopic approach. The surgical procedure involved the use of a Cavitron Ultrasonic Surgical Aspirator (CUSA™), Olympus Thunderbeat device, bipolar forceps, titanium clips, and endovascular staplers. Intraoperative ultrasonography was routinely performed to guide resection, and cholangiography was systematically used to detect potential biliary injuries. The Pringle maneuver was used in selected cases, depending on intraoperative bleeding risk and lesion location [20].

### 2.4. Data Collection

The main data required for evaluation, including ECOG performance status (PS), G8 geriatric score, MET (Metabolic Equivalent of Task) score, Charlson Comorbidity Index, American Society of Anaesthesiologists (ASA) physical status, Simplified Acute Physiology Score (SAPS II), and Sequential Organ Failure Assessment (SOFA) score, were recorded during the perioperative period. The type of cancer, the date of diagnosis, and its metastatic nature were also collected. Details of the surgical procedure, the anesthetic protocol, and intraoperative complications were recorded. Severe postoperative complications, including postoperative AKI, reoperation, or death, were recorded within 30 days of surgery, and all these data were classified according to the Dindo–Clavien classification. Therapeutic interventions during the IMC/ICU stay were also collected. Hospital lengths of stay were prospectively collected. Long-term survival was evaluated with a follow-up period of 1 year.

### 2.5. Study Outcomes

The primary outcome of the study was the incidence of postoperative AKI up to day 30 after surgery and associated risk factors. The secondary outcomes were the incidence of postoperative complications at day 30, 1-year mortality, and 1-year CKD incidence. For the latter two outcomes, the endpoints were 1-year survival and 1-year CKD-free survival, respectively.

### 2.6. Follow-Up

The patients were followed up for 1 year after the first day of surgery using the electronic system available at the hospital. The electronic system is used for administrative and medical purposes in all wards, and every procedure, visit, laboratory examination, vital sign, and other data gathered during hospitalization or outpatient visits are compulsorily recorded, along with the date and a unique identifier. The Paoli-Calmettes Institute has a policy of following up its patients, and, as a general rule, at least one visit is scheduled every 3 months during the first year for the postoperative follow-up of major oncological surgery. For patients lost to follow-up, we used the INSEE database to determine the status of deceased patients [21].

### 2.7. Statistical Analysis

All the data is presented as rates (percentages) for the qualitative variables and as medians [25th–75th percentiles] or means [standard deviations (SD)] for the quantitative variables. Data were compared between the following two groups of patients: the occurrence of AKI within the first 30 days of the postoperative period (AKI group) and no AKI during this period (no AKI group). Comparisons between the 2 groups of patients were realized using the Mann–Whitney test for continuous variables and the Chi-Square or Fisher’s exact tests for categorical variables. All *p* values < 0.05 were considered statistically significant. We performed a logistic regression analysis to identify independent variables associated with the development of postoperative AKI, as measured by the estimated odds ratio (OR) and 95% confidence interval (95% CI). Factors with significance or borderline significance (*p* < 0.1) in the univariate analyses and those related as pertinent factors in the literature were then included in a multivariable regression model with backward stepwise variable selection. We chose 0.1 as the critical *p* value for entry into the model and 0.1 as the *p* value for removal. The required significance level was set at a *p* value of <0.05. The Hosmer–Lemeshow test was used to check the goodness-of-fit of the selected logistic model. Based on β coefficients (log OR) obtained from the multivariate analysis, a predictive score for postoperative AKI occurrence was developed (the AKIMBO score). The receiver operating characteristic (ROC) curve for predicting the occurrence of AKI before day 30 was used to identify high-risk and low-risk patients, with the cut-off values defined based on the Youden index (sensitivity + specificity − 1). The following diagnostic performance parameters and their 95%CIs were calculated: sensitivity, specificity, predictive positive value (PPV), and negative predictive value (NPV). The discriminatory power of the predictive model was evaluated by computing the areas under the receiver operating characteristic curves (ROC-AUCs). Internal validation was realized using the bootstrap-corrected Harrell’s c-index (AUC) with 1000 replications. The bootstrap-corrected AUC and 95% confidence interval (CI) were reported.

A Cox proportional hazards model was used to evaluate the effect of AKI and other confounding variables on 1-year survival, with the results expressed as hazard ratios (HRs) and 95% confidence intervals [CIs]. One-year survival is also represented using Kaplan–Meier (KM) survival curves. Differences among different groups were evaluated using the log-rank test. We also analyzed the association of postoperative AKI with the occurrence of CKD within the first year following surgery. Since patients may die before the occurrence of CKD, we used a competing-risks analysis which accounted for the competing risk of death without CKD. CKD-free survival was defined as the interval between the date of surgery and the date of CKD occurrence, last follow-up, or death from any cause. For this endpoint, the follow-up period was censored at 12 months. Cumulative incidence curves were used to describe the cumulative incidence of CKD, and comparisons between groups were performed using Gray’s test. All tests were two-sided, and *p* values lower than 0.05 were considered statistically significant. Statistical analyses were performed with R statistical software, version 3.4.3 (available online at https://www.r-project.org/ accessed on 30 July 2025).

## 3. Results

**Figure 1:**
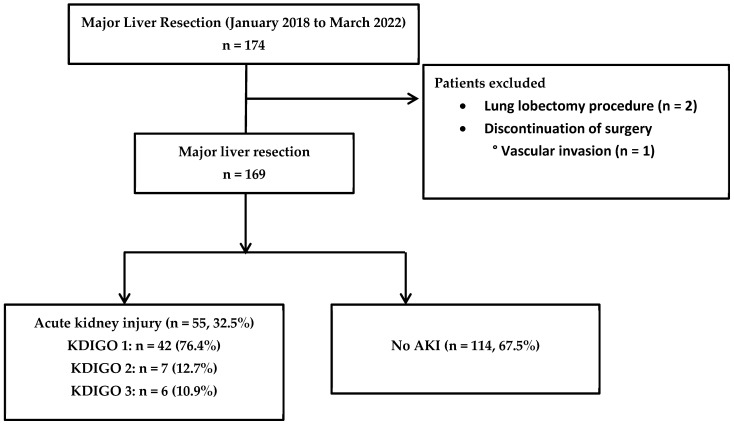
Study flow chart.

From January 2018 to March 2022, 169 patients were included in the study, as shown in Figure 1. Among them, 50.3% (n = 85) were male, the age was 67 (58–73) years, and the Charlson score was 8 (6–9). For these patients, the ASA score was two in 73.4% (n = 124) and three in 24.3% (n = 41) of cases. All patients were admitted postoperatively to the IMC/ICU for at least 48 h. Among them, 31.3% (n = 53) required postoperative vasopressor support and 6.5% (n = 11) underwent postoperative invasive mechanical ventilation. Fifty-five patients (32.5%) developed an AKI within 30 days follow surgery. These patients were classified as KDIGO stage 1 (n = 42, 76.4%), stage 2 (n = 7, 12.7%), and stage 3 (n = 6, 10.9%). Only one patient required continuous renal replacement therapy. Eighty percent of them (n = 44) developed AKI before day 4. Three patients developed AKI that persisted over time, meeting the definition of AKD. Fifty-two patients (98%) in the AKI group recovered normal renal function within 30 days. Patients developed severe postoperative complications (Dindo–Clavien Grade > II) in 25% (n = 42) of cases. Thirty-day mortality was 2.4% (n = 4) and 90-day mortality was 4.24% (n = 7), with four patients lost to follow-up. The median hospital length of stay was 10 (7–14) days. One-year mortality was 15.3% (n = 24), with 12 patients lost to follow-up.

### 3.1. Preoperative Period (Table 1)

During this period, the main factors associated with the occurrence of postoperative AKI were age (*p* = 0.001), sex (*p* < 0.001), cholangiocarcinoma (*p* < 0.001), preoperative treatment with ACE or ARB (*p* < 0.001), BMI (*p* < 0.001), ASA score (*p* < 0.001), and preoperative creatinine level (*p* = 0.003), while neoadjuvant chemotherapy (*p* = 0.019) and metastatic disease (*p* = 0.001) appeared to be protective. Charlson comorbidity index was not associated with the occurrence of postoperative AKI (*p* = 0.499). Among the 11 patients with cirrhosis, 10 were classified as Child–Pugh A, and classification was unavailable in 1 case due to missing data. This information has now been added to the revised manuscript. Among the 97 patients who received neoadjuvant chemotherapy, 86.3% (n = 88) had metastatic disease, whereas only 13.7% (n = 9) of non-metastatic cases received such treatment, *p* < 0.001).

### 3.2. Intraoperative Period (Table 2, Univariate Analysis)

During this period, the main factors associated with the occurrence of postoperative AKI were the use and dose of norepinephrine (*p* < 0.001), cumulative fluid intake (*p* < 0.001), fluid balance (*p* = 0.014), bleeding volume (*p* < 0.001), number of red blood cell units (*p* = 0.013), bile duct reconstruction (*p* = 0.027), intraoperative lactate (*p* = 0.043), and time to liver resection *p* = 0.046).

### 3.3. Multivariate Analysis

Using multivariate analysis, the factors independently associated with postoperative AKI were ACE or ARB (*p* = 0.021), age (*p* = 0.006), time to liver resection (*p* = 0.025), BMI (*p* = 0.028), and the use of intraoperative norepinephrine (*p* < 0.018), whereas neoadjuvant chemotherapy (*p* = 0.009) appeared to be a protective factor (Table 3).

### 3.4. Predictive Model

**Figure 2:**
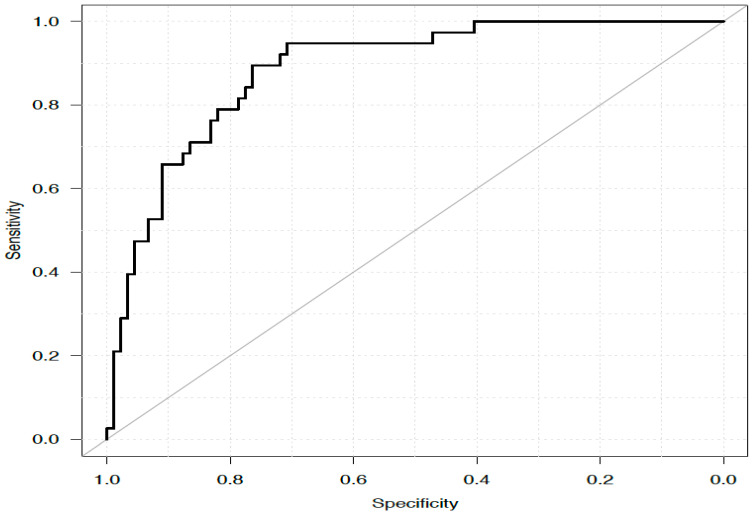
ROC curve of the AKIMEBO score.

Based on the β coefficients (log OR from the multivariate final model, Table 3), a predictive score for AKI (AKIMEBO score) was defined at the end of surgery, as follows: preoperative treatment with ACE or ARB (2 points), neoadjuvant chemotherapy (−2 points), bile duct reconstruction (2 points), age (0.1 points per year), time to liver resection (0.01 points per minute), the use of intraoperative norepinephrine (2 points), and BMI (0.2 points per point). At a cut-off value of ≥15.6, the AKIMEBO score was associated with an 89.5% sensitivity, 76.4% specificity, 61.8% PPV, and 94.4% NPV. The ROC-AUC was 0.885 (Figure 2). Internal validation using ROC analysis was performed in the same cohort. The bootstrap-corrected AUC of the predictive model was 0.882 (95% CI, 0.822–0.942). The effect of AKI incidence on the positive and negative predictive values using this cut-off value is displayed in Supplementary Figure S1. The graph in Supplementary Figure S2 illustrates how the sensitivity and specificity of the AKIMEBO score vary according to cut-off points.

### 3.5. Postoperative Period

During this period, on day 1, the SOFA and SAPS II scores were 4 [2–6] and 16 [11–24], respectively. Using the Dindo–Clavien classification, 38 patients (22.5%) presented Grade 2 complications, 15 (8.9%) presented Grade 3 complications, 23 (13.6%) presented Grade 4 complications, and 4 (2.4%) presented Grade 5 complications. The main complications were represented by ARF, which occurred in 50 patients (29.6%), with 11 patients needing invasive ventilation (6.5%). Infectious complications were also frequent (n = 42, 25%), as well as postoperative bleeding (n = 17, 10%). For this period, comparisons between AKI and non-AKI patients are shown in Table 4.

**Figure 3:**
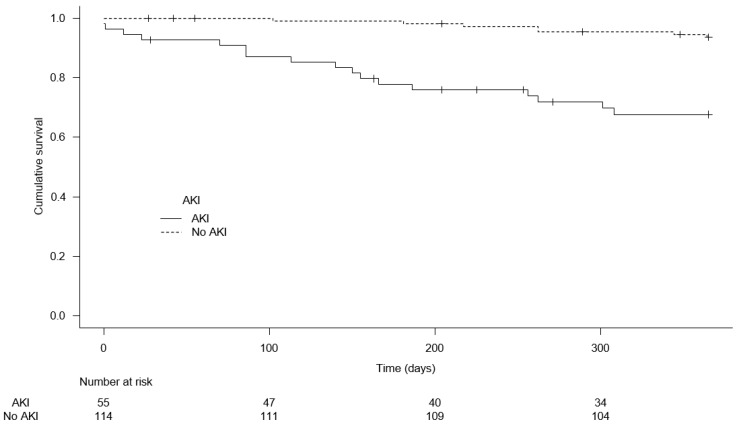
One-year survival according to AKI occurrence.

The occurrence of postoperative AKI was associated with 1-year mortality (HR = 6.29, 95%CI [2.60–15.19], *p* < 0.001). Figure 3 presents the Kaplan–Meier curve illustrating the impact of AKI on 1-year mortality (*p* < 0.001). The median 1-year follow-up was 351 days, 95% CI [342–360]. After adjustment for severity factors (SAPS II, vasopressors, renal replacement therapy, invasive mechanical ventilation, and metastasis), postoperative AKI was still associated with 1-year mortality (adjusted HR = 4.39, 95%CI [1.50–12.79], *p* = 0.007). Finally, at one year, nine patients (6%) developed CKD after the exclusion of patients with preoperative CKD (n = 10). AKI was not significantly associated with the occurrence of CKD at one year, Supplementary Figure S3 (*p* = 0.08 using Gray’s test).

## 4. Discussion

In this retrospective observational study, we report on 169 consecutive patients who underwent major hepatectomy over a 4-year period. In this study, the incidence of postoperative AKI was 32.5% after major hepatectomy. Pre- and intraoperative independent factors associated with the occurrence of postoperative AKI were BMI, age, preoperative treatment with ACE/ARB, the use of vasopressors during surgery, and time to liver resection. Neoadjuvant chemotherapy was protective. Based on the results of the multivariate model, we constructed a predictive score for postoperative AKI occurrence (the AKIMEBO score) with an NPV of 94.4%. Moreover, patients with postoperative AKI developed more major complications and had a higher one-year mortality.

In our study, the occurrence of AKI was found to be higher than the commonly reported 22% in the literature [4]. Several factors may explain this: we used the KDIGO definition, which appears to be more sensitive than the RIFLE or AKIN [22] definitions. In addition, we focused on the specific subset of major hepatectomies, generally known to be associated with postoperative complications [1,4,5].

Interestingly, patients who received neoadjuvant chemotherapy had a significantly better renal prognosis. This likely reflects a careful therapeutic selection, as these patients are typically considered fit for multimodal treatment strategies and benefit from structured preoperative optimization [23]. In our cohort, 86.3% of patients treated with neoadjuvant chemotherapy had metastatic disease, often allowing for more standardized and controlled surgical procedures. In contrast, cholangiocarcinoma was associated with a higher risk of postoperative AKI, likely due to more complex surgical approaches, frequently involving bile duct reconstruction, increased operative time, and greater intraoperative hemodynamic stress. The fact that bile duct reconstruction emerged as an independent risk factor for AKI further supports the idea that it may serve as a surrogate marker of cholangiocarcinoma-related complexity.

An important point in our study is the potential impact of systemic and renal hemodynamic changes on developing postoperative AKI. In fact, preoperative ACE/ARB treatment was associated with the occurrence of postoperative AKI. In non-cardiac surgery, the origin of postoperative AKI is multifactorial in patients with preoperative hypertension. Mechanisms involving reduced aortic compliance, microvascular changes due to mechanical pressure, and renal blood flow becoming highly pressure-dependent may be involved [24]. In this context, the association of postoperative AKI with preoperative ACE/ARB treatment could also be an indirect marker of hypertension rather than a real effect of these drugs. Indeed, in healthy subjects, GFR has been shown to be maintained until MAP falls below 80 mmHg. In patients with impaired autoregulation, specifically patients with arterial hypertension, there is a decrease in GFR at higher MAP values. This decrease is particularly noticeable when systolic blood pressure decreases or when patients are exposed to vasoconstrictive medications. This occurrence is commonly referred to as “normotensive acute ischemic renal failure” [25]. Along this line, the time elapsed before liver resection, which corresponds to the duration of a restricted vascular filling of 1 mL/kg/h, and the use of intraoperative vasopressors were relevant risk factors for AKI. Importantly, intraoperative fluid intake output, expressed as mL/kg/h, was similar in both patient groups at the end of surgery, while the duration of fluid restriction was longer in the AKI group than in the non-AKI group with similar post-hepatectomy fluid compensations. This suggests that fluid balance could not be effectively corrected at the end of surgery in the AKI group and failed to prevent the deleterious effects of prolonged reduced renal perfusion in this group of patients. An association between restrictive fluid intake, hypovolemia, acute tubular necrosis, and mortality during major surgery has recently been described [26,27]. Garnier et al. previously demonstrated that a hepatectomy duration of >250 min was predictive of severe acute renal failure [28,29]. Although intraoperative fluid restriction has shown benefits in terms of overall complications and a reduced length of stay [30,31], its use might represent a risk factor for AKI that should not be overlooked, since AKI was associated with 1-year mortality in our study. In this context, the lack of personalized protocols during hepatic resection time might lead to hepatic hypoperfusion, highlighting the need for prospective studies evaluating personalized protocols in major hepatectomy. These results suggest future avenues of research in the era of ERAS protocols, where fluid restriction is usually implemented during the whole period between the onset of anesthesia and the end of liver resection. Indeed, our results underscore the need for a more limited duration of fluid restriction, which could be circumscribed only around the time of liver transection. The ongoing OPTILIVER study (NCT04655885) should provide some clarification on this issue [32].

We showed that postoperative AKI was significantly associated with long-term mortality. Previous studies on abdominal surgery have already provided evidence of the detrimental effects of AKI on patient outcomes, which supports the consistency of our findings [4,7,33]. For instance, Tomozawa et al. demonstrated a correlation between AKI after liver resection surgery and various adverse outcomes, such as prolonged hospital stays, increased rates of artificial ventilation, reintubation, and the need for renal replacement therapy [7]. Similarly, Teixeira et al. found that postoperative AKI was independently associated with higher in-hospital mortality [34]. Out of the 55 patients diagnosed with AKI, 52 (95%) recovered renal function, while 3 (5%) developed AKD as a secondary condition to CKD. It is worth noting that among these 52 patients who recovered renal function within 30 days after experiencing AKI, 7 were diagnosed with CKD at one year. Despite the normalization of biological renal function within a month, there may still be underlying renal damage, leading to the development of CKD in the long term. Interestingly, we did not find a statistically significant association between the incidence of AKI and CKD despite the extensive literature on the topic. Animal models have demonstrated abnormal repair of the tubular epithelial barrier following severe AKI, resulting in interstitial fibrosis and progressive CKD [35,36]. However, it is possible that our study lacked sufficient statistical power to detect such an association.

Given its potential long-term negative effects, it is important to accurately predict the risk of acute kidney injury (AKI) during the intraoperative stage. Previous studies have attempted to predict AKI occurrence after liver surgery. Kim et al. conducted a retrospective multicenter study involving 4325 patients who had undergone liver surgery. They developed a predictive score that could be calculated intraoperatively, which had an AUC of 0.71 [8]. Similarly, Slankemenac conducted a single-center study aiming to support decision making. Their model achieved a better accuracy with an AUC of 0.79; however, the definition of AKI used was based on the AKIN definition and the study population differed, potentially consisting of fewer patients with comorbidities [29]. Postoperative AKI is a high-risk situation, and when this complication can be anticipated, at-risk patients could be managed in the ICU. With an NPV of 94%, our proposed AKIMBO score would have made it possible to avoid systematic admission to the ICU, where resources are limited, for most patients with a score of < 15.6. On the other hand, considering the incidence of AKI (90% within the first 96 h) and the PPV of 62%, a score of ≥15.6 could induce hospitalization in the ICU in 6 out of 10 patients with a proven risk of developing AKI, while for 4 out of 10 patients, this hospitalization could be excessive, but interrupted after 4 days in the absence of AKI. This score should be confirmed in a multicenter validation cohort.

Several limitations must be acknowledged. First, our sample size was relatively small, which may have limited the statistical power of our results. As a result, we may have missed some important risk factors that have already been identified in the literature. Second, it is important to note that our study focused on a specific population subgroup—patients undergoing major hepatectomy according to the ERAS protocol. Consequently, our results may not be generalizable to all patients undergoing hepatectomy. The specific protocols and practices used in our study may not be representative of those in other hospital settings or patient populations. Third, the AKIMEBO score has not yet been externally validated. Although internal validation using bootstrap resampling demonstrated a good discriminatory performance, further prospective multicenter studies are needed to assess its external validity and clinical generalizability. Finally, the retrospective design of the study may present several pitfalls.

## 5. Conclusions

In conclusion, AKI following major hepatectomy is a common complication with an early onset and potential resolution. However, our study underscores the significance of AKI as a predictor of one-year mortality and its strong association with short-term outcomes. Although our study found a non-significant trend between AKI and CKD, it highlights the value of seven predictive factors for AKI occurrence. From these factors, the AKIMEBO score can be employed intraoperatively to help clinicians identify patients at a lower risk of developing AKI. The implementation of the AKIMEBO score holds promise for facilitating the selection of postoperative clinical pathways for patients undergoing major hepatectomy. By focusing on individualized intraoperative protocols for blood pressure management and volume expansion, physicians can address the two key areas for improvement in preventing AKI. These findings provide valuable insights for optimizing patient care and outcomes in the context of major hepatectomy.

## Figures and Tables

**Table 1:** Preoperative characteristics, univariate analysis.

	All (n = 169)	No AKI (n = 114)	AKI (n = 55)	*p* Value
Age (years)	71 [58.00–73.00]	65 [57.00–70.75]	69 [65.00–74.50]	0.001
Male sex (%)	85 (50.3)	45 (39.5)	40 (72.7)	<0.001
ASA score (%)				<0.001
ASA 1	4 (2.37)	3 (2.6)	1 (1.8)	
ASA 2	124 (73.37)	94 (82.5)	30 (54.5)	
ASA 3	41 (24.26)	17 (14.9)	24 (43.6)	
Comorbidities (%)				
Malnutrition	39 (23.08)	26 (22.8)	13 (23.6)	1.00
History of hypertension	78 (46.15)	33 (28.9)	35 (63.6)	<0.001
Diabetes mellitus	25 (14.79)	12 (11.5)	13 (23.7)	0.036
COPD	20 (11.83)	9 (7.9)	11 (20.0)	0.043
Coronary heart disease	12 (7.10)	6 (5.3)	6 (10.9)	0.308
Systolic heart failure	1 (0.59)	1 (0.9)	0 (0.0)	1.00
Cirrhosis	11 (6.51)	5 (4.4)	6 (10.9)	0.201
Scores				
Charlson comorbidity index	8.5 [6.00–9.00]	8.00 [6.00–9.00]	8.00 [5.50–9.00]	0.499
MELD score	6.00 [6.00–7.00]	6.00 [6.00–7.00]	7.00 [6.00–8.00]	0.002
LEE score				0.026
0	144 (85.21)	102 (89.5)	42 (76.4)	
1	20 (11.83)	11 (9.6	9 (16.4)	
2	5 (2.96)	1 (0.9)	4 (7.3)	
>3	0 (0.00)	0 (0.00)	0 (0.00)	
Treatments				
Curative anticoagulation	20 (11.83)	9 (7.9)	11 (20)	0.043
Anti-aggregation therapy	24 (14.20)	10 (8.8)	14 (25.5)	0.007
ACE inhibitors/AR blockers	43 (25.44)	16 (14)	27 (49.1)	<0.001
Thiazide diuretics	15 (8.88)	6 (5.3)	9 (16.4)	0.037
Furosemide	3 (1.78)	0 (0.00)	3 (5.5)	0.058
Beta blocker drugs	26 (15.38)	12 (10.5)	14 (25.5)	0.022
Calcium channel blockers	25 (14.79)	14 (12.3)	11 (20.0)	0.274
Statins	26 (15.38)	12 (10.5)	14 (25.5)	0.022
Oral antidiabetics	18 (10.65)	7 (6.1)	11 (20.0)	0.013
Neoadjuvant chemotherapy	97 (57.40)	73 (64)	24 (43.6)	0.019
Preoperative chemo/radioembolization	20 (11.83)	9 (7.9)	11 (20.4)	0.038
Preoperative portal embolization	86 (50.89)	51 (44.8)	35 (63.6)	0.022
Renal status GFR (mL/min/1.73 m^2^)				0.015
>90	102 (60.36)	78 (68.4)	24 (44.4)	
60–90	56 (33.14)	33 (28.9)	23 (42.6)	
45–60	7 (4.14)	2 (1.8)	5 (9.3)	
30–45	3 (1.78)	1 (0.9)	2 (3.7)	
<30	0 (0.00)	0 (0.0)	0 (0.00)	
Preoperative biology				
Hemoglobin (g/dL)	13.0 [12.2–14.0]	12.9 [12.2–14.0]	13.4 [11.8–14.4]	0.792
Albumin (g/L)	39.0 [35.1–42.0]	39.50 [36.0–42.0]	38.1 [35.0–41.0]	0.267
Creatinine (μmol/L)	66.0 [57.0–79.0]	64.75 [56.1–74.8]	72.0 [61.0–94.0]	0.003
Bilirubin (μmol/L)	8.3 [6.2–11.6]	8.05 [6.1–10.3]	10.0 [6.3–12.8]	0.083
Main liver tumors				
Metastatic cancer	102 (60.3)	79 (69.3)	23 (41.8)	0.001
Hepatocellular carcinoma	22 (13)	11 (9.6)	11 (20.4)	0.093
Cholangiocarcinoma	28 (16.7)	10 (8.8)	18 (32.7)	<0.001

Data are presented in median [quartiles] and n (percentages). ASA, Physical status score from American Society of Anesthesiologists; COPD, Chronic Obstructive Pulmonary Disease; ERAS, Enhanced Recovery After Surgery; MELD, Model for End-Stage Liver Disease; ACE, Angiotensin-Converting Enzyme; ARB, Angiotensin II Receptor Blockers; GFR, Glomerular Filtration Rate.

**Table 2:** Intraoperative characteristics, univariate analysis.

	No AKI (n = 114)	AKI (n = 55)	*p* Value
Surgical procedures			
Open surgery	100 (87.7)	53 (96.4)	0.129
Laparoscopic surgery	22 (19.3)	5 (9.1)	0.141
Pringle maneuver	34 (29.8)	13 (23.6)	0.511
Inferior vena cava clamping	6 (5.4)	4 (7.4)	0.603
Bile duct reconstruction	11 (9.6)	13 (23.6)	0.027
Vascular procedure	17 (14.9)	10 (18.2)	0.685
Duration of procedures			
Anesthesia (min)	487 [429–570]	550 [471–640]	0.002
Surgical (min)	393 [312–472]	434 [361–531]	0.019
Pringle maneuver	0.00 [0.00–10.00]	0.00 [0.00–0.00]	0.450
Inferior vena cava clamping	0.00 [0.00–0.00]	0.00 [0.00–0.00]	0.670
Time to liver resection	397 [310–472]	426 [368–501]	0.046
Vasopressors			
Norepinephrine use	6 (5.3)	21 (38.2)	<0.001
Norepinephrine cumulative dose (ug)	0.00 [0.00–271.15]	407.05 [0.00–3192.50]	<0.001
Fluid parameters			
Cumulative fluid intake (mL)	3470 [2717–4165]	4460 [3445–6140]	<0.001
Fluid intake output (mL/kg/h)	6.37 [5.47–8.04]	6.17 [5.39–8.24]	0.846
Urine output (mL/kg/min)	0.80 [0.52–1.19]	0.55 [0.33–0.84]	<0.001
Fluid balance (mL)	2097 [1341–2874]	2636 [1797–3934]	0.014
Bleeding			
Volume (mL)	300 [200–500]	600 [350–800]	<0.001
RBCs units	0.00 [0.00–0.00]	0.00 [0.00–0.00]	0.013
Lactate kinetic within the first 24 h			
Peak lactate level on day 0 (mmol/L)	3.40 [2.30–4.70]	4.50 [2.90–5.70]	0.043
Peak lactate level on day 1 (mmol/L)	3.65 [2.50–5.10]	4.50 [2.90–5.68]	0.165
Lactate clearance (day 0–day 1) (mmol/L)	0.00 [0.00–0.79]	0.00 [−0.007–0.17]	0.031
Nephrotoxic drugs			
Aminoglycosides	14 (12.3)	5 (9.1)	0.722
NSAIs	91 (79.8)	41 (74.5)	0.563

Data are presented in median [quartiles] and n (percentages). RBCs, Red Blood Cells; NSAI, Non-Steroidal Anti-Inflammatory drugs.

**Table 3:** Multivariate logistic regression of potential pre- and intraoperative AKI predictors.

Variables	OR	*p* Value	95% CI	β (log OR)
Preoperative treatment with ACE/ARB	5.914	0.021	1.31 to 26.70	1.777
Neoadjuvant chemotherapy	0.144	0.009	0.03 to 0.61	−1.936
Bile duct reconstruction	5.538	0.088	0.77 to 39.61	1.712
Age (per year)	1.114	0.006	1.03 to 1.20	0.108
Time to liver resection (per min)	1.008	0.025	1.01 to 1.16	0.008
Intraoperative use of vasopressors	8.663	0.018	1.44 to 51.84	2.159
Body Mass Index (kg/m^2^) (per point)	1.239	0.028	1.02 to 1.50	0.214

ACE. Angiotensin-Converting Enzyme; ARBs. Angiotensin II Receptor Blockers.

**Table 4:** Postoperative characteristics.

	No AKI (n = 114)	AKI (n = 55)	*p* Value
SAPS II	25 [19–30]	32 [24–40]	<0.001
SOFA day 1	3 [2–5]	6 [4–7]	<0.001
SOFA day 3	2 [0–2]	2 [2–4]	<0.001
Fluid parameters (from day 0 to day 1)			
Cumulative fluid intake (mL)	4410 [3490–5650]	6910 [4870–9270]	<0.001
Fluid balance (mL)	3365 [2136–4415]	5580 [3595–7692]	<0.001
Renal function on day 90			
GFR (mL/min/1.73 m^2^)			0.033
>90	55 (62.5)	15 (38.5)	
60–90	30 (34.1)	20 (51.3)	
45–60	2 (2.3	2 (5.1)	
30–45	0 (0.0)	2 (5.1)	
<30	1 (1.1)	0 (0.00)	
Renal replacement therapy	0 (0.0)	1 (1.8)	0.709
Nephrotoxic Drugs			
NSAIs	92 (81.4)	28 (51.9)	<0.001
Ketoprofen cumulative dose in IMC/ICU	200 [100.00–400.00]	50.00 [0.00–200.00]	<0.001
Postoperative complications up to day 30			
Sepsis	19 (16.7)	23 (41.8)	0.001
Vasopressors	24 (21.1)	29 (52.7)	<0.001
ARF	25 (21.9)	25 (45.5)	0.003
Oxygen therapy	24 (21.1)	24 (43.6)	0.004
Non-invasive mechanical ventilation	2 (1.8)	6 (10.9)	0.025
Invasive mechanical ventilation	1 (0.9)	10 (18.2)	<0.001
PHLF	2 (1.8)	2 (3.8)	0.338
Surgical re-operation	5 (4.4)	9 (16.4)	0.019
Total RBC units	0.00 [0.00–0.75]	0.00 [0.00–3.00]	0.001
Dindo–Clavien stage			<0.001
I or no complication	73 (64)	16 (29.1)	
II	28 (24.6)	10 (18.2)	
III	8 (7.0)	7 (12.7)	
IV	5 (4.4)	18 (32.7)	
V	0 (0.0)	4 (7.3)	
Hospital length of stay (days)	8.00 [7.00–12.00]	13.00 [8.00–16.50]	<0.001
Thirty-day mortality	0 (0.0)	4 (7.3)	0.018

Data are presented in median [quartiles] and n (percentages). GFR, Glomerular Filtration Rate; ICU, Intensive Care Unit; ARF, Postoperative Acute Respiratory Failure; PHLF, Post-Hepatectomy Liver failure; RBCs, Red Blood Cells; SOFA, Sepsis-related Organ Failure Assessment; SAPS II, Simplified Acute Physiology Score II; NSAI, Non-Steroidal Anti-Inflammatory Drugs.

## Data Availability

Data cannot be shared publicly because consent for publication of raw data was not obtained from study participants. Data are available from the Internal Review Board (IRB) of Institut Paoli Calmettes (contact via S.Maick, MAICKS@ipc.unicancer.fr) for researchers who meet the criteria for access to confidential data.

## Supplementary Materials

The following supporting information can be downloaded at: https://www.mdpi.com/article/10.3390/jcm14155452/s1, Figure S1: Effect of AKI incidence on NPV and PPV values; Figure S2: (Cumulative distribution analysis (CDA): Sensitivity and specificity against the cutoff values; Figure S3: Impact of AKI on CKD incidence.

## Abbreviations

AKI, Acute Kidney Injury; AKD, Acute Kidney Disease; CKD, Chronic Kidney Disease; ERAS, Enhanced Recovery After Surgery; ICU, Intensive Care Unit; IMC, Intermediate Care Unit; ASA, American Society of Anesthesiologists (Physical Status Classification); SAPS II, Simplified Acute Physiology Score II; SOFA, Sequential Organ Failure Assessment; GFR, Glomerular Filtration Rate; CKD, Chronic Kidney Disease; MAP, Mean Arterial Pressure; BMI, Body Mass Index; KDIGO, Kidney Disease: Improving Global Outcomes; PHLF, Post-Hepatectomy Liver Failure; GLIM, Global Leadership Initiative on Malnutrition; COPD, Chronic Obstructive Pulmonary Disease; RBC, Red Blood Cells; NSAI, Non-Steroidal Anti-Inflammatory; ARF, Acute Respiratory Failure; IMV, Invasive Mechanical Ventilation; NIV, Non-Invasive Ventilation; ACE, Angiotensin-Converting Enzyme (inhibitors); ARB, Angiotensin II Receptor Blockers; AUC, Area Under the Curve (of ROC); ROC, Receiver Operating Characteristic; OR, Odds Ratio; HR, Hazard Ratio; CI, Confidence Interval; PPV, Positive Predictive Value; NPV, Negative Predictive Value; MET, Metabolic Equivalent of Task; ECOG PS, Eastern Cooperative Oncology Group Performance Status; AKIMEBO, Acute Kidney Injury Model based on multivariate predictors
