# Supplementary material for: Incidence and Predictive Factors of Acute Kidney Injury After Major Hepatectomy: Implications for Patient Management in Era of Enhanced Recovery After Surgery (ERAS) Protocols
Source: J Clin Med. 2025 Aug 2;14(15):5452. doi: 10.3390/jcm14155452 (PMC12347576; doi:10.3390/jcm14155452)

Supplementary file S1: Effect of AKI incidence on NPV and PPV values

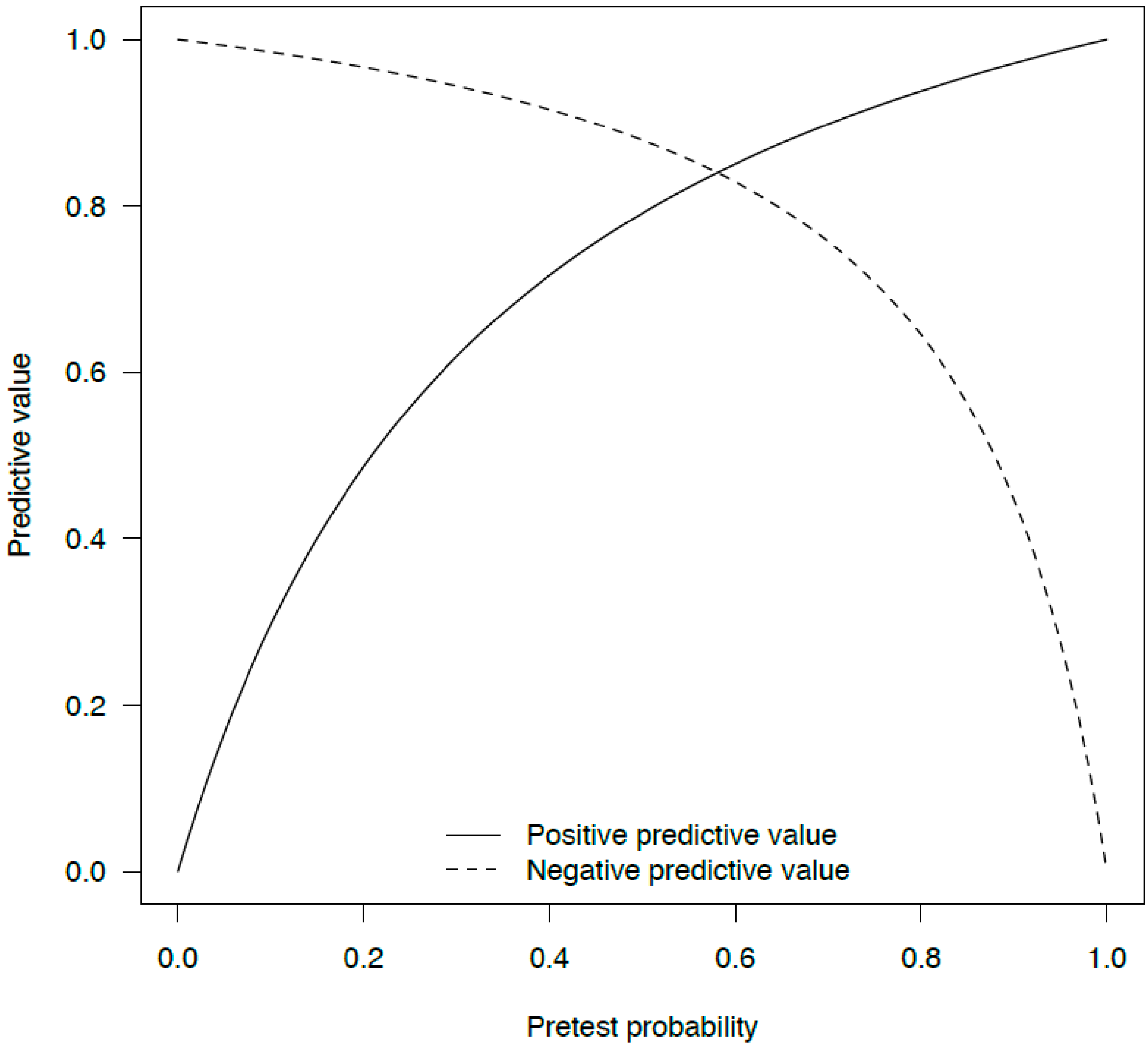

Supplementary file S2 (Cumulative distribution analysis (CDA): Sensitivity and specificity against the cutoff values

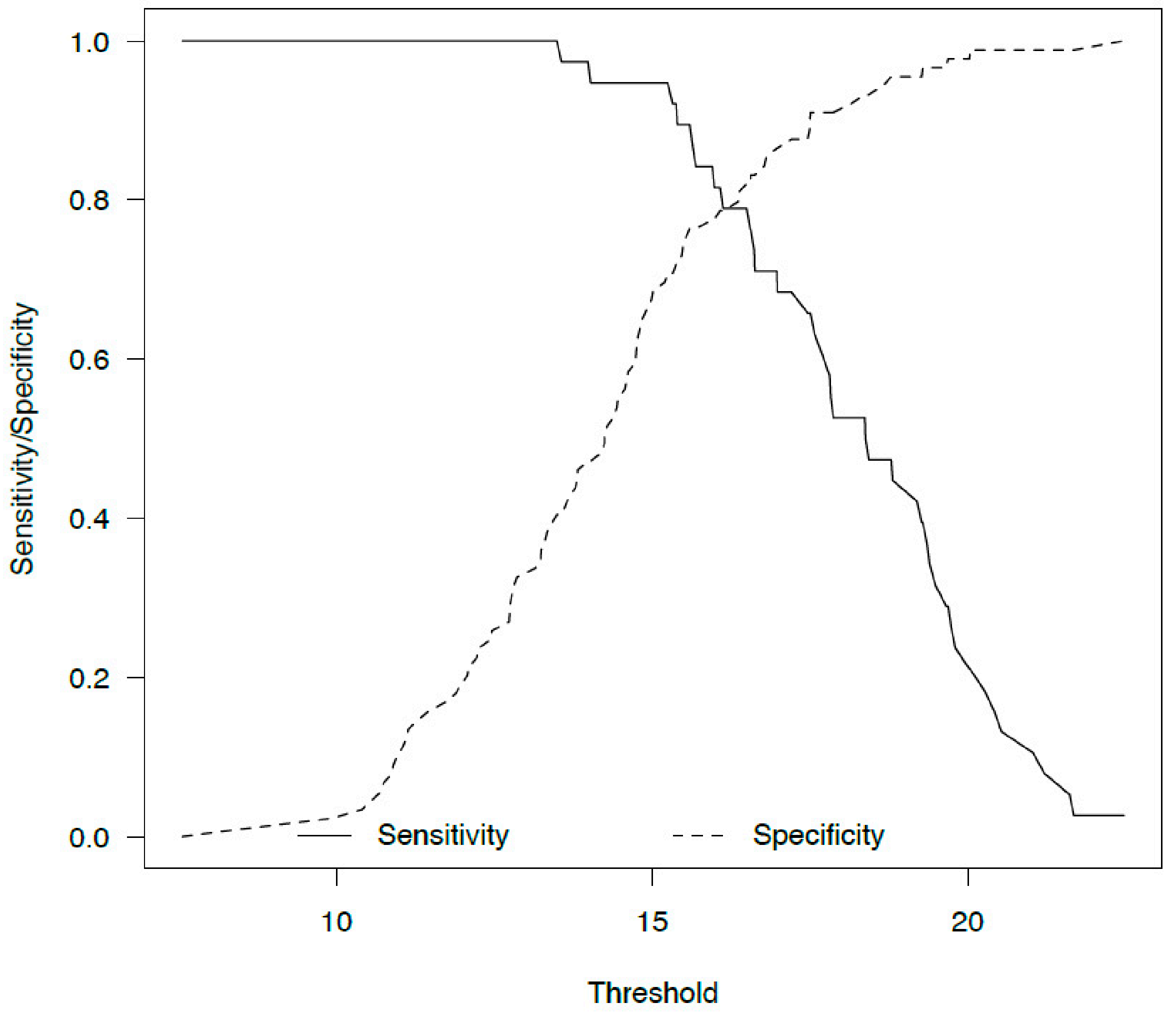

Supplementary file S3: Impact of AKI on CKD incidence.

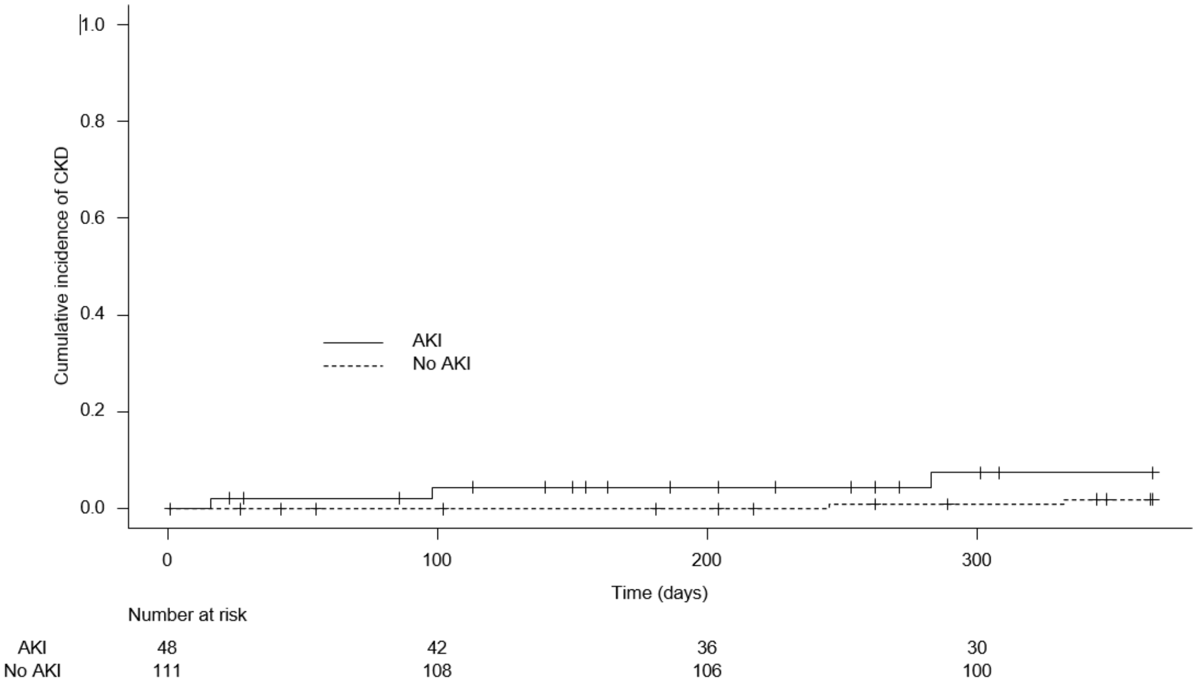

Supplement: Supplementary file 1 [file jcm-14-05452-s001.zip › jcm-3764423-supplementary.pdf]
